# Response of Photosynthesis and Chlorophyll Fluorescence to Nitrogen Changes in Rice with Different Nitrogen Use Efficiencies

**DOI:** 10.3390/plants14101465

**Published:** 2025-05-14

**Authors:** Zexin Qi, Chen Xu, Rui Tang, Qiang Zhang, Wenzheng Sun, Chenglong Guan, Ye Wang, Mengru Zhang, Jiale Ding, Yuankai Zhang, Hong Yang, Ying Yang, Xiaolong Liu, Zhian Zhang, Fenglou Ling

**Affiliations:** 1 Agronomy College, Jilin Agricultural University, Changchun 130118, China; 2 Institute of Agricultural Resources and Environment, Jilin Academy of Agricultural Sciences, Changchun 130033, China; 3 College of Life Science and Resources and Environment, Yichun University, Yichun 336000, China

**Keywords:** rice, chlorophyll, chlorophyll fluorescence, low nitrogen tolerance, photosynthesis

## Abstract

Nitrogen is a key element in promoting crop growth and development and improving photosynthesis. This study aimed to study the response of two rice genotypes to the restoration of N supply after varying periods of N deficiency. We used the low-nitrogen-tolerant rice Jijing 88 (JJ 88) and the nitrogen-sensitive rice variety Xinong 999 (XN 999) as test materials. The results of this study indicated that, compared to XN 999, JJ 88 has a higher content of the photosynthetic pigments. Photosynthesis in JJ 88 has strong adaptability under low-nitrogen conditions. Upon an increase in the nitrogen supply level, the maximum regeneration rate of ribulose biphosphate (RuBP, *J_max_*) and the maximum carboxylation rate of RuBP (*V_cmax_*) in JJ 88 showed a relatively large increase. The chlorophyll fluorescence parameters, including the effective quantum yield of photosystem II (Φ_PSII_), the efficiency of excitation capture by open PSII centers (Fv′/Fm′), photochemical fluorescence quenching (qP), and the electron transfer rate (ETR) decreased slightly, while the non-photochemical fluorescence quenching (NPQ) increased slightly. Under low-nitrogen conditions, low-nitrogen-tolerant rice varieties maintain reasonable growth during the seedling stage. With an increase in the nitrogen supply level, the dry matter accumulation, photosynthetic pigment content, photosynthesis, and electron transfer ability of plants improve, but not to normal nitrogen supply levels. However, compared with XN 999, JJ 88 has a more proactive recovery ability. The research results provide valuable guidance for the breeding of nitrogen-efficient rice varieties and nitrogen fertilizer management.

## 1. Introduction

Rice is one of the most important cereal crops in the world and serves as the main food for more than half of the world’s population [1]. Investigating the physiological and anatomical determinants of rice photosynthesis, along with the plant’s response to environmental stimuli, is crucial for enhancing rice yield. The photosynthesis process in C_3_ plants, such as rice, is constrained by their capacity for CO_2_ diffusion and the efficiency of their biochemical functions [2,3,4,5,6]. Before being processed by Rubisco’s key enzymes in the Calvin cycle, CO_2_ from the atmosphere first diffuses through the stomata to reach the substomatal cavity. Subsequently, it diffuses through the cell wall, plasma membrane, cytoplasm, and chloroplast envelope to arrive at the carboxylation site [7,8]. Through an in-depth understanding of the biochemical mechanism of leaf photosynthesis, it was possible to establish a model for the response of leaf photosynthesis to the light intensity and CO_2_ concentration [9,10]. According to the model created by Farquhar et al. [10], the ability of ribulose 1,5-bisphosphate carboxylase/oxygenase (Rubisco) to consume ribulose biphosphate (RuBP carboxylation), limitations in the electron transport, and the ability of Calvin cycle enzymes to regenerate RuBP (RuBP regeneration) affect CO_2_ assimilation rates in C_3_ plants. Based on this model, there were two main factors that limited the rate of CO_2_ assimilation in C_3_ plants: (1) the balance between the maximum regeneration rate of RuBP (*J_max_*) and the maximum carboxylation rate of RuBP (*V_cmax_*); (2) CO_2_ concentration in chloroplasts [11]. Grassi et al. [12] showed that the ability of *Picea abies* Karst seedlings to adapt to changes in nutrient supply can be determined based on the changes in intercellular CO_2_ and chlorophyll fluorescence parameters. Research has shown that at the same reference temperature, the higher the nitrogen content of leaves is, the stronger the photosynthetic capacity is [13]. Some studies have reported greater allocation of nitrogen to Rubisco and free amino acids under high nitrogen availability [14,15,16]. Moderate N nutrition can ensure the normal biosynthesis of photosynthetic pigments and enhance the ability of light energy capture and conversion, improving photochemical efficiency in plant leaves [16,17]. Leaves’ photosynthetic rate depends on their nitrogen nutritional status and environmental conditions [18]. There is ample experimental evidence that demonstrates increases and decreases in photosynthesis during the ontogenesis of plant tissue. However, factors such as light, temperature, and the environment can affect photosynthesis [19]. While the temperature is the key determinant of *V_cmax_* and *J_max_*, the moisture and nutrient availability also have important effects on these processes. Changes in plants’ photosynthetic productivity in response to their growing environment may be related to the ecological conditions under which rice is grown, such as field management practices, nutrient application rates, and endogenous differences between species/genotypes [19,20]. In fact, some studies have reported strong correlations between photosynthetic capacity and leaf N and P concentrations [18,21]. In many higher plant species, the photosynthetic capacity of leaves is related to their nitrogen content, as most of the nitrogen in leaves is present in matrix enzymes and thylakoid proteins. Studies [22,23] have shown that *V_cmax_* and *J_max_* also increase as the N content in leaves increases. Therefore, under nitrogen-limited conditions, CO_2_ assimilation is affected during long-term growth due to carbohydrate accumulation and a source–sink imbalance [24]. The net photosynthetic rate (A) versus the intercellular CO_2_ concentration (Ci) curve was used to study the photosynthetic behavior and assimilation response of A to an increased CO_2_ concentration. Under initial conditions, when Ci was low and the RuBP supply was in a saturated state, the change in A depended on *V_cmax_*. As Ci further increased, A’s dependence shifted to *J_max_* due to a sufficient CO_2_ supply [25,26]. In C_3_ plants, nearly 25–40% of leaf nitrogen is used for Rubisco synthesis, and the large amount of nitrogen input in Rubisco will lead to a decrease in nitrogen use efficiency [27]. However, few studies have investigated the potential effects of nitrogen on photosynthetic parameter responses, and the mechanisms controlling this impact are still unclear. Although a few studies have attributed the decreased CO_2_ assimilation in plants under N limiting conditions to stomatal conductance [28], most of the previous works suggest that a decline in the biochemical capacity for carboxylation and damaged photosystem II (PSII), revealed by the decreased activity of Rubisco and the diminished maximal efficiency of PSII photochemistry (Fv/Fm), respectively, are the key constraining factors for photosynthesis in plants under N-limiting conditions. Furthermore, photochemical and non-photochemical quenching can help plants to grow under low-N conditions [28]. Chlorophyll fluorescence parameters reflect the light energy conversion efficiency, electron transfer rate (ETR), and energy dissipation rate of photosystem II (PS II) in plants [29]. To understand the physiological state of a specific plant and assess the photosynthetic damage resulting from environmental stress, chlorophyll fluorescence serves as a sensitive tool for detecting plant adaptability to environmental changes and elucidating the photosynthetic strategies of various species. Chlorophyll fluorescence exhibits significant variations under different environmental conditions. The light energy that is absorbed by chlorophyll molecules can be directed in three primary ways: to initiate photosynthesis, to dissipate as heat, or to be emitted as fluorescence [30]. The photosynthetic efficiency of PS II under light conditions (ΔF/Fm′) and under dark adaptation (Fv/Fm) are among the most commonly utilized chlorophyll fluorescence measurement parameters in plant research [29]. Under adverse conditions, additional energy is dissipated in the form of heat or fluorescence [31]. Non-photochemical quenching (NPQ) is a self-protection mechanism for plants to dissipate excess light energy. This is because plants typically absorb more light energy than they use in photosynthesis. Research has shown that stress conditions increase NPQ, while reducing both the effective quantum yield of PS II (Φ_PSII_) and fluorescence quenching (qP) [31,32,33]. Lin et al. [34] showed that nitrogen application increased the Fv/Fm, Φ_PSII_, ETR, and qP of naked oats. Other studies have shown that nitrogen deficiency reduces the Fv/Fm and Φ_PSII_ of coffee and corn [35,36]. There are obvious differences in the growth response of different rice varieties to nitrogen. Numerous studies have shown that nitrogen deficiency has different effects on dry matter production and leaf photosynthesis in different rice varieties [37,38,39].

A prolonged low nitrogen supply or subsequent increase in nitrogen supply can affect plant photosynthesis. However, there are few studies on the effects of nitrogen deficiency and subsequent increase in nitrogen supply on photosynthesis and chlorophyll fluorescence at different growth stages of rice varieties with different nitrogen use efficiencies. This study used low-nitrogen-tolerant rice and nitrogen-sensitive rice varieties as materials, and used hydroponics throughout the growth period. The effects of nitrogen deficiency and compensation at different growth stages on the dry matter accumulation, photosynthesis, carbon dioxide reaction, chlorophyll content, and chlorophyll fluorescence-related parameters were explored. This provides a theoretical basis for the breeding of low-nitrogen-tolerant rice varieties and nitrogen fertilizer management.

## 2. Results

### 2.1. Chlorophyll Content

**Figure 1:**
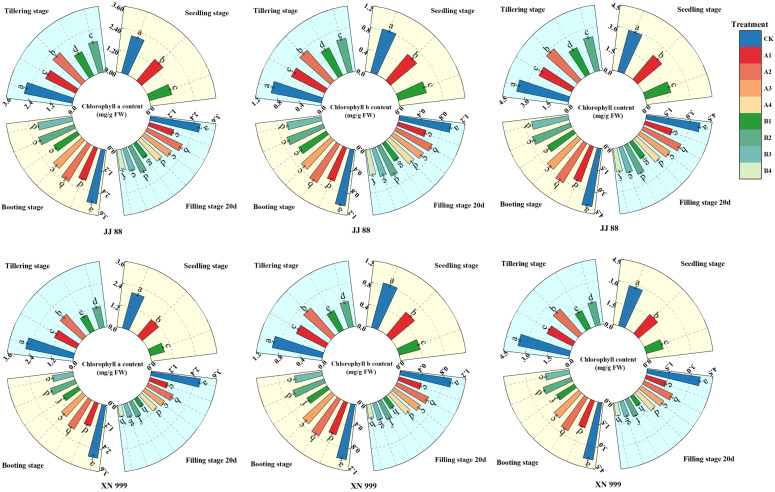
The effects of nitrogen concentration on Cha, Chb, and Chl contents in JJ 88 and XN 999. Values are presented as means ± SD, *n* = 3. Different letters on the columns represent significant differences (*p* < 0.05) between different treatments of the same rice varieties based on Duncan’s test.

Compared with the control treatment (CK), the low-nitrogen treatment reduced the chlorophyll content in leaves. As nitrogen supply increased, the trends in leaf chlorophyll content differed between JJ 88 and XN 999 (Figure 1). Throughout the growth period, the contents of Cha, Chb, and Chl first increased and then decreased. As the nitrogen supply level was restored, the increase ranges of Cha, Chb, and Chl contents in JJ 88 and XN 999 under low-nitrogen treatment were different. During the tillering stage, JJ 88 showed no significant differences in the contents of Cha, Chb, and Chl under the A1 and B2 treatments. During the booting stage, JJ 88 exhibited no significant differences in Cha, Chb, and Chl contents under the A3 and B2 treatments, nor under the A1 and B3 treatments. XN 999 also exhibited no significant differences in Cha, Chb, and Chl contents under the A1 and B2 treatments. At filling stage 20 d, JJ 88 showed no significant differences in Cha, Chb, and Chl contents under the A1 and B3 treatments, nor under the A4 and B2 treatments. XN 999 exhibited no significant differences in Cha, Chb, and Chl contents under the B1 and B4 treatments.

### 2.2. Parameters Related to Photosynthesis

**Figure 2:**
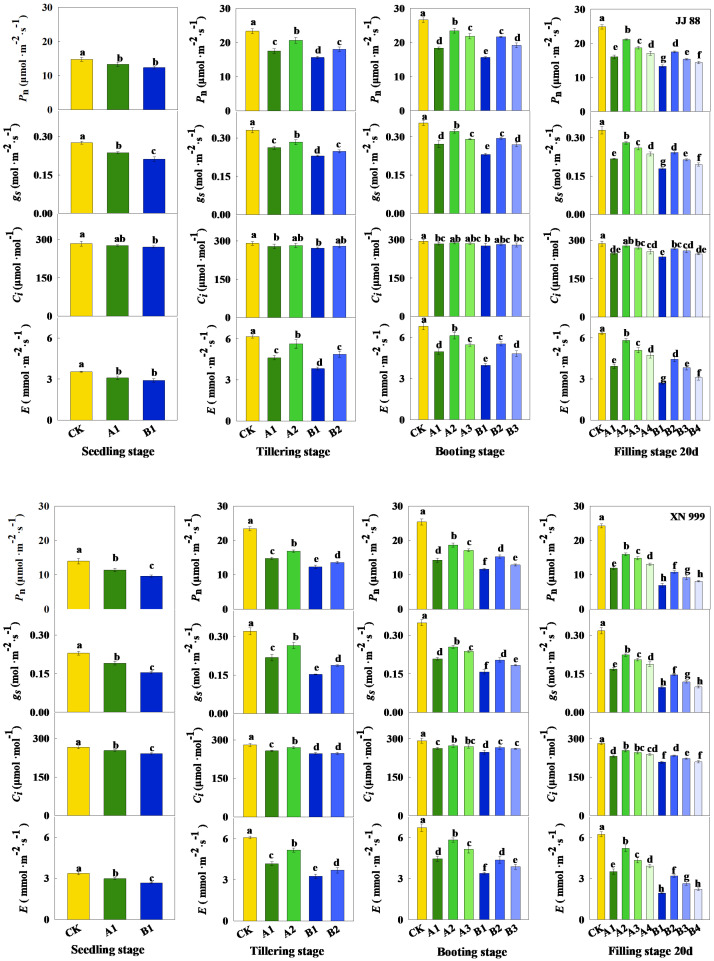
The effects of nitrogen concentration on Pn, gs, Ci, and E in JJ 88 and XN 999. Values are presented as means ± SD, *n* = 3. Different letters on the columns represent significant differences (*p* < 0.05) between different treatments of the same rice varieties based on Duncan’s test.

The effects of different treatments on the photosynthetic parameters of rice leaves varied, mainly affecting the net photosynthesis (*P*_n_), stomatal conductance (*g*_s_), and transpiration rate (*E*) (Figure 2). During the seedling stage, there was no significant difference in *P*_n_ and *E* in JJ 88 under the A1 and B1 treatments. During the tillering stage, JJ 88 showed no significant differences in *P*_n_, *g*_s_, and *E* under the A1 and B2 treatments. During the booting stage, JJ 88 did not show any significant differences in *P*_n_, *g*_s_, and *E* under the A1 and B3 treatments, nor under the A3 and B2 treatments. XN 999 show no significant differences in *P*_n_, *g*_s_, and *E* under the A1 and B2 treatments. At filling stage 20 d, JJ 88 exhibited no significant differences in *P*_n_, *g*_s_, and *E* under the A1 and B3 treatments, nor under the A4 and B2 treatments. XN 999 exhibited no significant differences in *P*_n_, *g*_s_, and *E* under the B1 and B4 treatments.

### 2.3. Carbon Dioxide Response Parameters

**Figure 3:**
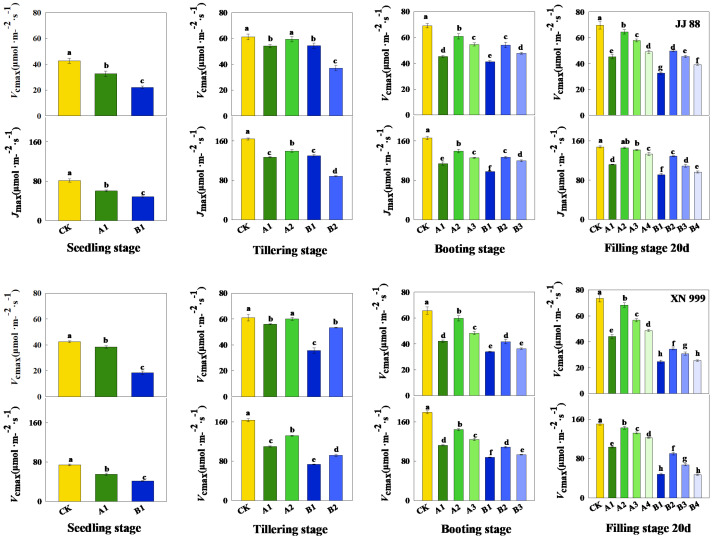
The effects of nitrogen concentration on Jmax and Vcmax in JJ 88 and XN 999. Values are presented as means ± SD, *n* = 3. Different letters on the columns represent significant differences (*p* < 0.05) between different treatments of the same rice varieties based on Duncan’s test.

After the nitrogen supply level increased, the rice’s *J_max_* and *V_cmax_* increased (Figure 3). Throughout the entire growth period, the *J_max_* and *V_cmax_* of the rice show a trend of first increasing and then slightly decreasing. During the tillering stage, there was no significant difference in the *J_max_* and *V_cmax_* of JJ 88 under the A1 and B2 treatments. During the booting stage, there was no significant difference in the *J_max_* and *V_cmax_* of JJ 88 under A3 and B2 treatments, nor was there a significant difference under the A1 and B3 treatments. *J_max_* and *V_cmax_* of XN 999 showed no significant difference under the A1 and B2 treatments. At filling stage 20 d, there was no significant difference in the *J_max_* and *V_cmax_* of JJ 88 under the A1 and B3 treatments, nor was there a significant difference under the A4 and B2 treatments. The *J_max_* and *V_cmax_* of XN 999 showed no significant difference under the B1 and B4 treatments.

### 2.4. Chlorophyll Fluorescence Parameters

**Figure 4:**
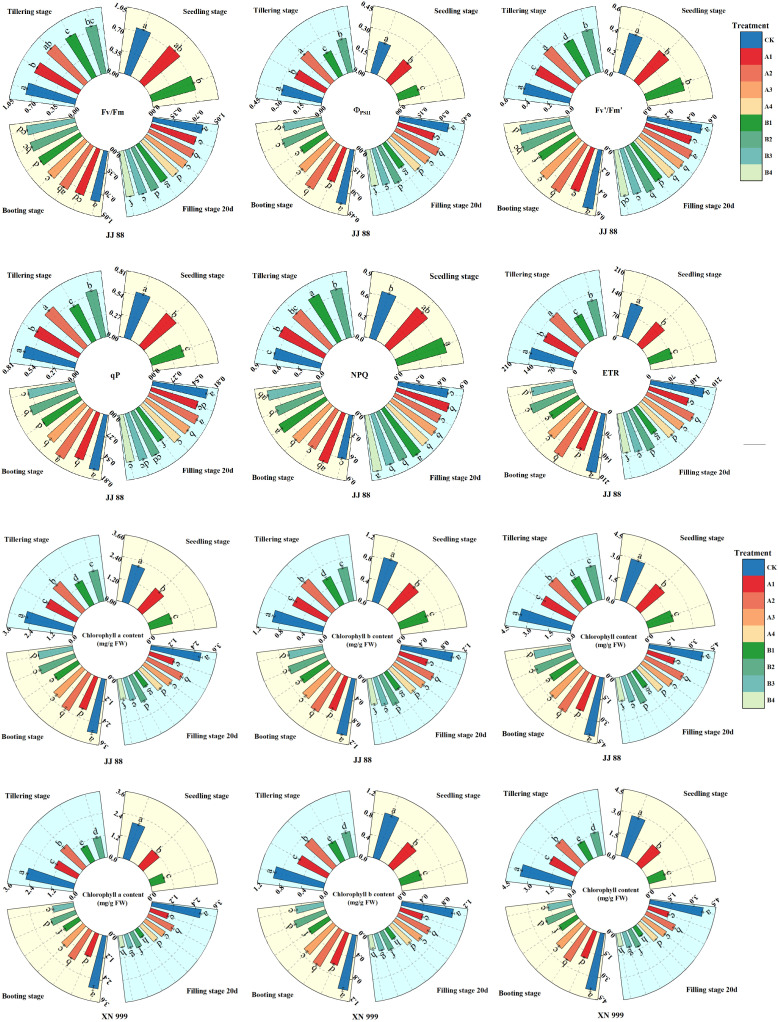
The effects of nitrogen concentration on the Fv/Fm, ΦPSII, Fv′/Fm′, qP, NPQ, and ETR of JJ 88 and XN 999. Values are presented as means ± SD, *n* = 3. Different letters on the columns represent significant differences (*p* < 0.05) between different treatments of the same rice varieties based on Duncan’s test.

It can be seen from Figure 4 that the changes in chlorophyll fluorescence parameters varied under different treatments. After the nitrogen supply level was increased, the Fv/Fm, Φ_PSII_, Fv′/Fm′, qP, and ETR increased, while NPQ decreased. During the processing, the Φ_PSII_, Fv′/Fm′, qP, and ETR showed a trend of first increasing and then decreasing, with Fv/Fm and NPQ showing relatively small changes over time. At the tillering stage, there were no significant differences in the Fv/Fm, Φ_PSII_, qP, NPQ, and ETR of JJ 88 under treatments A1 and B2, while the Φ_PSII_, NPQ, and ETR of XN 999 were significantly different under different treatments. During the booting stage, there were no significant differences in the Fv/Fm, Φ_PSII_, Fv′/Fm′, qP, NPQ, and ETR of JJ 88 under the A3 and B2 treatments; meanwhile, there were no significant differences in the Fv/Fm, Φ_PSII_, NPQ, and ETR under treatments A1 and B3. There were no significant differences in the Φ_PSII_, Fv′/Fm′, qP, NPQ, and ETR of XN 999 under treatments A1 and B2. At filling stage 20 d, there were no significant differences in the Fv/Fm, Φ_PSII_, Fv′/Fm′, qP, NPQ, and ETR of JJ 88 between the A1 and B3 treatments, nor between the A4 and B2 treatments. There were no significant differences in the Fv/Fm, Φ_PSII_, qP, and NPQ of XN 999 between the B1 and B4 treatments.

### 2.5. Dry Matter and Nitrogen Accumulation

**Figure 5:**
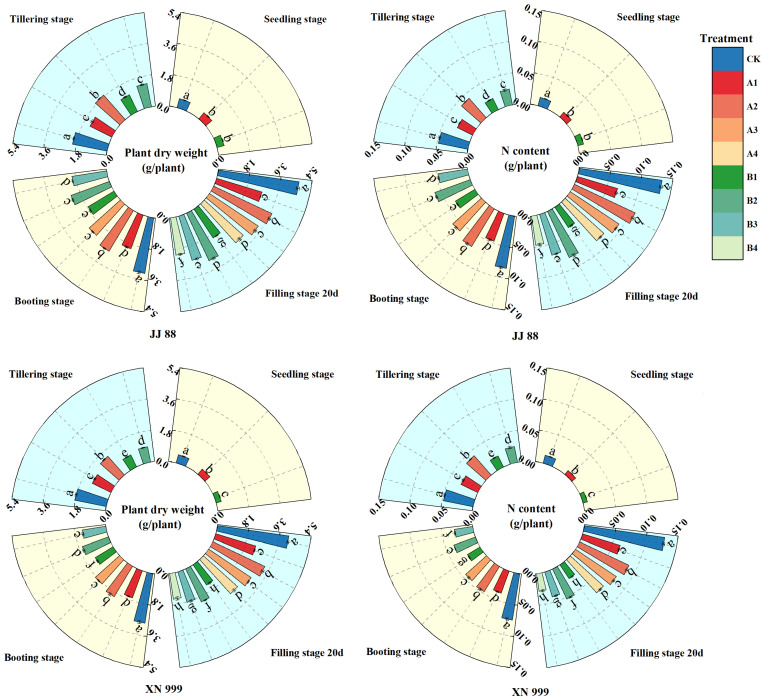
The effect of nitrogen concentration on dry matter accumulation (DW) and nitrogen accumulation (NC) in JJ 88 and XN 999. Values are presented as means ± SD, *n* = 3. Different letters on the columns represent significant differences (*p* < 0.05) between different treatments of the same rice varieties based on Duncan’s test.

As shown in Figure 5, the accumulation of dry matter (gDW/plant) and nitrogen (gN/plant) in rice showed an upward trend over time. As the nitrogen supply increased, rice dry matter and nitrogen accumulation increased, but the changes were different in JJ 88 and XN 999. During the seedling stage, the nitrogen-sensitive rice exhibited significant differences in dry matter and nitrogen accumulation across various treatments. At the tillering stage, no significant differences were observed in the dry matter and nitrogen accumulation of JJ 88 between treatments A1 and B2. Similarly, at the booting stage, there were no significant differences in the dry matter and nitrogen accumulation of JJ 88 under treatments A1 and B3, nor between treatments A3 and B2. Additionally, no significant differences in dry matter accumulation of XN 999 were noted between treatments A1 and B2. At filling stage 20 d, there were no significant differences in the dry matter and nitrogen accumulation of JJ 88 between the A1 and B3 treatments, nor the A4 and B2 treatments. Furthermore, no significant differences in dry matter and nitrogen accumulation were found for XN 999 under the B1 and B4 treatments.

### 2.6. Correlation Analysis

**Figure 6:**
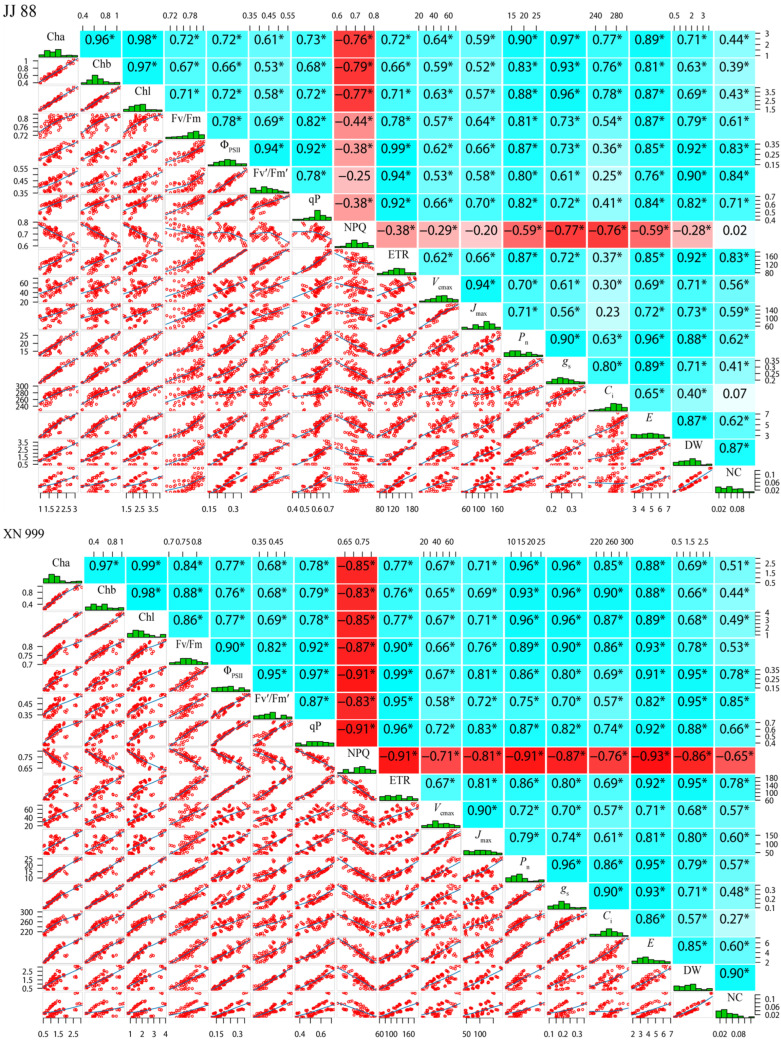
Correlation analysis of photosynthesis, fluorescence, dry matter, and nitrogen accumulation in JJ 88 and XN 999. Note: *—significant difference at the 0.05 level (*p* < 0.05). The red background of the data in the figure shows negative correlation, while the blue background shows positive correlation.

Figure 6 shows the correlation between the chlorophyll content, chlorophyll fluorescence parameters, carbon dioxide response parameters, photosynthetic parameters, dry matter, and nitrogen accumulation in rice. There was a significant negative correlation between the NPQ of JJ 88 and the Cha, Chb, Chl, Fv/Fm, Φ_PSII_, qP, ETR, *V_cmax_*, *P*_n_, *g*_s_, *C*_i_, *E*, and DW. The DW and NC were significantly positively correlated with the Cha, Chb, Chl, Fv/Fm, Φ_PSII_, Fv′/Fm′, qP, ETR, *V_cmax_*, *J_max_*, *P*_n_, *g*_s_, and *E*. XN 999’s NPQ demonstrated a significant negative correlation with the Cha, Chb, Chl, Fv/Fm, Φ_PSII_, Fv′/Fm′, qP, ETR, *V_cmax_*, *J_max_*, *P*_n_, *g*_s_, *C*_i_, *E*, DW, and NC, while the DW and NC showed significant positive correlations with the Cha, Chb, Chl, Fv/Fm, Φ_PSII_, Fv′/Fm′, qP, ETR, *V_cmax_*, *J_max_*, *P*_n_, *g*_s_, *C*_i_, and *E*.

### 2.7. Redundancy Analysis

**Figure 7:**
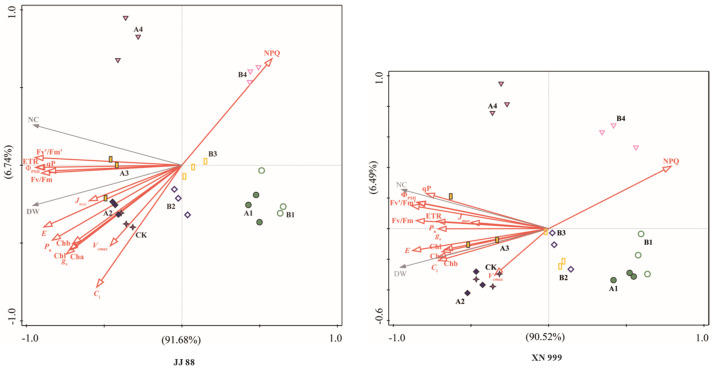
Redundancy analysis of photosynthesis, fluorescence, dry matter, and nitrogen accumulation in JJ 88 and XN 999.

According to Figure 7, the RDA1 and RDA2 axes together explain 98.42% of the responses of dry matter and nitrogen accumulation to photosynthetic fluorescence in JJ 88, while the RDA1 and RDA2 axes together explain 97.01% of the responses of dry matter and nitrogen accumulation to photosynthetic fluorescence in XN 999. This shows that the RDA results are reliable, and the dry matter and nitrogen accumulation of these rice varieties are closely related to the photosynthetic fluorescence characteristics. The dry matter and nitrogen accumulation of JJ 88 and XN 999 are negatively correlated with the photosynthetic fluorescence parameter NPQ, and positively related to the Fv/Fm, Φ_PSII_, Fv′/Fm′, qP, ETR, *P*_n_, *g*_s_, *C*_i_, *E*, Cha, Chb, Chl, *V_cmax_*, and *J_max_*.

## 3. Discussion

Photosynthesis is the fundamental physiological process through which plants convert solar energy into chemical energy. While photosynthesis requires light, excessive absorption of light energy by pigment molecules can disrupt the oxidative balance within the plant, thereby inhibiting the process, particularly in C_3_ plants [40]. Chlorophyll is the main component of light-harvesting compounds and light reaction centers, and increasing its content enhances the capture and absorption of light energy [41]. Leaf chlorophyll a and b act as the main light-absorbing and transmitting pigments (antenna pigments), improving the light capture efficiency and thereby increasing the net photosynthetic rate of plants. The presence of nitrogen enhances the ability of plants to carry out photosynthesis, which may be due to an increase in chlorophyll, a key molecule for capturing light energy [42]. In this study, the contents of Cha, Chb, and Chl first increased and then decreased over time. After the nitrogen supply level increased, the Cha, Chb, and Chl contents increased, but the increases in Cha, Chb, and Chl contents in JJ 88 and XN 999 were different. At filling stage 20 d, there was no significant difference in the Cha, Chb, and Chl contents in XN 999 under the B1 and B4 treatments, but there was a significant difference in JJ 88. This indicates that, compared with XN 999, JJ 88 can maintain a higher chlorophyll content under low-nitrogen conditions and that the increase in chlorophyll content is greater with increasing nitrogen concentrations, with significant differences between different treatments over time.

On the other hand, the process of photosynthesis requires a system of numerous proteins. Nitrogen in plants mainly accumulates in leaves, and up to three-quarters of leaf nitrogen is invested in photosynthetic organs, representing the largest nitrogen pool in plants [43,44,45]. Leaf photosynthesis is largely controlled by the nitrogen supply levels. It is this nitrogen demand that builds the photosynthetic system, resulting in the need to consume large amounts of nitrogen fertilizer during crop production [46]. Therefore, reducing the application of nitrogen fertilizer in agricultural production without reducing the yield is also crucial for the sustainable development of agriculture [47]. The results of this study indicate that low-nitrogen conditions reduce *g*_s_ and *C*_i_ in rice leaves and affect *P*_n_ and *E*. At the tillering stage, booting stage, and filling stage 20 d, an increased nitrogen supply enhanced the photosynthetic performance of JJ 88 significantly more than that of XN 999, which was mainly reflected in changes in *P*_n_, *g*_s_, and *E*. *J_max_* and *V_cmax_* are considered the main driving factors of photosynthesis. Research has shown that the *g*_s_ of leaves is closely related to *J_max_* and *V_cmax_,* indicating that the movement of gases through the stomata is related to the efficiency of the photosynthetic process [48]. In this study, as the nitrogen supply level is restored, the *g*_s_, *J_max_* and *V_cmax_* of rice leaves all increase. However, the increase ranges of JJ 88 and XN 999 are different. To sum up, JJ 88 still showed a large increase. The changes in chlorophyll content further illustrate that as the nitrogen supply level increases, JJ 88’s ability to capture light energy increases, thereby improving the photosynthetic capacity of the leaves.

Chlorophyll fluorescence is emitted by higher plants and reflects the complex activities of photosynthesis. The Fv/Fm, Φ_PSII_, Fv′/Fm′, qP, NPQ, and ETR are representative chlorophyll fluorescence parameters that are widely utilized to study the effects of environmental stress on plants. Rice cannot achieve an optimal photosynthetic rate under low nitrogen conditions, and the imbalance between light absorption and utilization renders it vulnerable to photoinhibition. This imbalance can be influenced by various factors, including the chlorophyll content and NPQ. NPQ serves as a photoprotective mechanism that dissipates excess irradiance energy. Studies have shown that stress immediately increases NPQ, which is critical for reducing electron transfer, enhancing heat dissipation, and ultimately improving rice’s resistance to abiotic stress [49]. Research has also shown that low-nitrogen treatment inhibits the photosynthesis and growth of rice and significantly enhances the induction of NPQ [50]. Chen and Cheng [51] studied the response of grapes to N treatment. The results showed that, compared with grapes with sufficient nitrogen, nitrogen deficiency made grapes more sensitive to light-induced damage, primarily manifested by an increased induction of NPQ [51]. These studies have confirmed that excess excitation in the photosystem leads to a reduction in light absorption and enhancement of NPQ induction. This enhanced NPQ further illustrates the degree of nitrogen deficiency in rice. In this study, low-nitrogen treatment induced an increase in NPQ at various growth stages, and NPQ decreased with an increase in nitrogen concentration. The NPQ of JJ 88 and XN 999 remained relatively high under low-nitrogen treatment. As the nitrogen supply level increased, the NPQ in JJ 88 decreased strongly. This shows that JJ 88 has stronger adaptability after increasing the nitrogen supply level, a weaker inhibitory effect on light, and an enhanced ability to resist strong light damage.

The PSII photochemical maximum quantum yield (Fv/Fm) increased slowly with the treatment time, but there were differences under different treatments. In the early stage of the low-nitrogen treatment, the primary photochemical reaction of PSII was not greatly affected. As the treatment time progressed, the primary photochemical reaction of PSII was inhibited, resulting in a decrease in Fv/Fm. At filling stage 20 d, there was no significant difference in the Fv/Fm of JJ 88 under the A1 and B3 treatments, or the A4 and B2 treatments. There was no significant difference in the Fv/Fm of XN 999 under the A1, A3, and A4 treatments or under the B1 and B4 treatments. After low-nitrogen treatment, the Fv/Fm of JJ 88 and XN 999 were decreased. As the nitrogen supply level increased, JJ 88 showed a greater trend of increasing over time. These findings suggest that JJ 88 possesses a robust capacity to safeguard the PSII reaction center from damage. A decrease in Φ_PSII_ indicates potential damage to the PSII reaction center. Furthermore, the parameter qP reflects the proportion of PSII antenna pigments that are effectively absorbing light energy for photochemical electron transfer [52]. Studies have shown that increasing nitrogen application increases Φ_PSII_ and qP, which helps to increase the photochemical effective quantum yield of PSII. In this study, compared with XN 999, the values of Φ_PSII_ and qP of JJ 88 were larger under low-nitrogen conditions. After the nitrogen supply level was restored, the values of Φ_PSII_ and qP of JJ 88 increase greatly with the nitrogen level. This indicates that the photosynthetic machinery’s ability to maintain QA in an oxidized state was enhanced, leading to an increase in the proportion of “open“ PSII reaction centers. This further confirms that low-nitrogen-tolerant rice varieties exhibit good adaptability when the nitrogen supply levels are increased. The application of the appropriate amount of N could increase the solar energy conversion efficiency in the PSII reaction center of rice leaves by improving the electron transfer efficiency and enhancing electron flow [53,54]. The ETR primarily reflects the electron transport dynamics occurring within the PSII reaction center, which can be quantified by the number and rate of absorbed light quanta throughout the electron transport process [55]. In this study, low-nitrogen treatment reduced the ETR. The research results of this experiment are consistent with previous studies. The reduction in *P*_n_ inhibited electron transfer and light energy conversion. The changing trends of *P*_n_ and the ETR in the test further verified this conclusion [56]. Under low-nitrogen conditions, the ETR of genotype JJ 88 was higher than that of genotype XN 999. This indicated that JJ 88 has a good electron transfer ability under low-nitrogen conditions compared with XN 999. Fv′/Fm′ reflects the excitation energy capture efficiency of the PSII reaction centers. After low-nitrogen treatment, Fv′/Fm′ decreased slightly, but the increase in Fv′/Fm′ was greater in JJ 88 with increasing nitrogen supply levels. This shows that the excitation energy of JJ 88 can be transferred to the open PSII reaction center more efficiently.

Nitrogen affects the metabolic level, physiological characteristics, and biomass synthesis of plants, mainly affecting the accumulation of dry matter [57,58,59]. The results showed that with the increase in nitrogen supply level, the adaptability of JJ 88 was stronger than that of XN 999. The changes in the dry matter accumulation of JJ 88 and XN 999 were different under different treatments. At filling stage 20 d, there was a significant difference in dry matter accumulation in JJ 88 under the B1 and B4 treatments, while there was no significant difference in XN 999. The reason for this difference may be that JJ 88 accumulates more nitrogen under low nitrogen conditions, so more nitrogen is used to accumulate dry matter [60,61]. In this study, the nitrogen accumulation and dry matter trends of rice plants were consistent, and increased with the increase in nitrogen supply, which is consistent with previous research results [62,63].

## 4. Materials and Methods

### 4.1. Plant Materials

The plant materials are provided by the Rice Research Institute of Jilin Agricultural University. Among them, the Jijing 88 (JJ 88) rice variety has a strong low-nitrogen tolerance, while the Xinong 999 (XN 999) rice variety has a weak low-nitrogen tolerance. The screening method of low-nitrogen-tolerant rice varieties can be found in Qi et al. [64].

### 4.2. Experimental Design

The experiment was conducted in the greenhouse of Jilin Agricultural University in 2023, utilizing natural lighting. The seeds were disinfected with 0.5% sodium hypochlorite for 10 min. After disinfection, the residual sodium hypochlorite was removed from the surface of the rice seeds using distilled water, and the seeds were subsequently placed in a culture dish for germination. Germinated seeds were then sown in vermiculite, with three seeds being planted per hole. The seedling tray was positioned within the seedling box and kept in water for five days. Following this, the seedlings were transferred to a half-concentration Kimura B nutrient solution for a duration of seven days. Subsequently, the nutrient solution was changed to an improved Kimura B formulation, utilizing NO_3_^−^ and NH_4_^+^ as nitrogen sources for treatment. Three nitrogen concentration treatments were established: the nutrient solution with NO_3_^−^ (1.6 mM) and NH_4_^+^ (1.6 mM) was labeled as 1N; the solution with NO_3_^−^ (0.8 mM) and NH_4_^+^ (0.8 mM) was labeled as 1/2N; and the solution with NO_3_^−^ (0.4 mM) and NH_4_^+^ (0.4 mM) was labeled as 1/4N.

**Figure 8:**
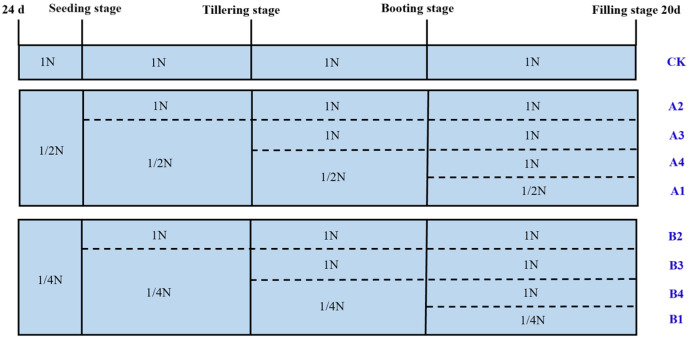
Schematic diagram of processing in different periods.

The 1/2N and 1/4N treatments were changed to the 1N treatment on the 21st day of treatment, the tillering stage, and the booting stage, respectively (Figure 8). Measurements and samples were taken at 45 d of growth (seedling stage), and at the tillering stage, booting stage, and filling stage 20 d. There were a total of nine treatments in this experiment: CK (1N supply), A1 (1/2N supply), A2 (1/2N supply restored to 1N supply during seedling stage), A3 (1/2N supply restored to 1N supply during tillering stage), A4 (1/2N supply restored to 1N supply during booting stage), B1 (1/4N supply), B2 (1/4N supply restored to 1N supply during seedling stage), B3 (1/4N supply restored to 1N supply during tillering stage), and B4 (1/4N supply restored to 1N supply during booting stage). The nutrient solution was replaced every three days and adjusted to pH 5.5 using HCl and NaOH. The concentrations of H_2_PO_4_^−^, K^+^, Ca^2+^, Mg^2+^, Na^+^, and trace elements in the nutrient solution remains unchanged, among which Fe is replaced by Fe (EDTA-Na_2_).

### 4.3. Measurement Indicators and Methods

#### 4.3.1. Dry Matter Determination

After seedling stage, tillering stage, booting stage, and filling stage 20 d, the rice plants were dried at 85 °C to a constant weight.

#### 4.3.2. Determination of Photosynthetic Parameters

Using a portable photosynthesis measurement system (Li-6400, LI-COR Inc., Lincoln, NE, USA), the photosynthesis-related parameters (*P*_n_, *g*_s_, *C*_i_, *E*) of the fully unfolded top leaves were measured from 8:00 am to 11:00 am on sunny days. The light intensity in the leaf chamber was set to 1200 μmol·m^−2^s^−1^. Three rice plants with similar growth conditions were selected for each treatment.

Chlorophyll fluorescence parameters were measured using a Li-6800 portable photosynthetic measurement system (LI-COR Inc. Lincoln, NE, USA) equipped with a 6800-01A fluorescent leaf chamber. The temperature was measured according to the actual temperature setting in open top chamber, the CO_2_ concentration in the leaf chamber was set to 400 μmol·mol^−1^, and fully unfolded leaves were selected. The fluorescence index measurement under a light adaptation environment was measured between 09:00 and 11:30 am, while under dark adaptation conditions, it was measured from 20:00 to 22:00 at night. The light intensity in the leaf chamber was set to 1200 μmol·m^−2^s^−1^. The calculation formulae for chlorophyll fluorescence-related parameters are as follows: Fv/Fm = (Fm − Fo)/Fm; F′v/F′m = (F’m − F′o)/F′m; Φ_PSII_ = (F′m − Fs)/F′m; qP = (F′m − Fs)/F′m − F′o; NPQ = (Fm − F′m)/F′m; ETR = 0.84 × PAR × Φ_PSII_/2. Here, PAR stands for photosynthetically active radiation.

#### 4.3.3. Carbon Dioxide Response Parameter Measurement

Using the LI-6800 portable photosynthetic measurement system (LI-COR Inc., Lincoln, NE, USA), the measurement site and period were the same as those for photosynthetic parameter measurement. A near-saturating photosynthetic photon flux density of 1400 μmol·m^−2^s^−1^ from an inbuilt LI-6800 LED light source was maintained. According to CO_2_ concentrations of 400, 350, 300, 250, 200, 150, 100, 50, and 25 μmol·mol^−1^, 0, 400, 400, 600, 800, 1000, 1200, 1400, 1600, 1800, and 2000 μmol·mol^−1^ sequentially determine the net photosynthetic rate. The fitaci function of “planteophis” (R packages) was used to fit the CO_2_ response curve. Based on the measurement data from the A-Ci curve of the FvCB model, fitting was performed using the fitaci function to estimate the maximum Rubisco regeneration rate (*J_max_*) and maximum carboxylation rate (*V_cmax_*).

#### 4.3.4. Chlorophyll Content Determination

An amount of 0.1 g of fresh leaves was weighed and soaked in a 10 mL mixture of equal volumes of ethanol and acetone in for 24 h in the dark. The extract was diluted and the absorbance was measured at 663 nm, 645 nm, and 470 nm, and the chlorophyll content was calculated [65].

Chlorophyll a = 12.21A663 − 2.81A645; chlorophyll b = 20.13A645 − 5.03A663; carotenoids = (1000A470 − 3.27Ca − 104Cb)/229; total pigment content = Ch a + Ch b.

#### 4.3.5. Nitrogen Content Determination

After curing at 105 °C for 30 min, the plant matter was dried at 80 °C and weighed. The weighed stems and leaves were directly crushed then sieved through an 80 mesh sieve, and the sample was digested with concentrated H_2_SO_4_-H_2_O_2_. The total nitrogen content was determined using the micro Kjeldahl nitrogen determination method [65].

Nitrogen accumulation = Plant dry matter mass × Plant nitrogen content

### 4.4. Statistical Analysis

Excel 2021 software was used for preliminary collation and analysis of data. SPSS 23.0 software was used to analyze the data. Based on a one-way analysis of variance (ANOVA), Duncan’s multiple range test (DMRT) was used to compare differences in the means among treatments. The significance level was *p* < 0.05. Origin 2021 and SigmaPlot 14.0 software were used for plotting, the R language 4.4.3 software “gpairs” package was used for correlation analysis and plotting, and Canoco 5 software was used for the redundancy analysis.

## 5. Conclusions

This experiment investigated the effects of nitrogen levels on the dry matter and N accumulations, photosynthesis, carbon dioxide response, chlorophyll, and chlorophyll fluorescence-related parameters of low-nitrogen-tolerant rice and nitrogen-sensitive rice varieties. Compared with the control treatment, the low-nitrogen treatment had an inhibitory effect on rice growth. This inhibitory effect was related to a decrease in chlorophyll content and a decrease in *P*_n_ caused by changes in *g*_s_, *E*, and chlorophyll fluorescence. However, there were differences in these changes between JJ 88 and XN 999. After the nitrogen supply level increased, the Cha, Chb, and Chl contents of JJ 88 tended to increase more than those of XN 999; the ability to enhance photosynthesis was strong, mainly manifested in increases in *P*_n_, *g*_s_, and *E*. The increases in the carbon dioxide response parameters *J_max_* and *V_cmax_* also verified the improvement in photosynthetic capacity. The decreases in the chlorophyll fluorescence parameters Φ_PSII_, Fv′/Fm′, qP, and ETR were relatively small, as was the increase in NPQ. Dry matter and nitrogen accumulated more. After the low-nitrogen treatment, JJ 88 exhibited a strong growth ability in the seedling stage, which provided a solid foundation for rice growth. Over time, as the nitrogen supply increased, JJ 88 became more adaptable to nitrogen, and the degree of damage to the rice plant’s growth, photosynthesis, carbon dioxide response, chlorophyll, and chlorophyll fluorescence weakened. In summary, the tolerance ability of rice to low nitrogen conditions not only shows differences in dry matter accumulation and nitrogen accumulation, but also shows differences in photosynthesis and chlorophyll fluorescence levels.

## Data Availability

The original contributions presented in the study are included in the article; further inquiries can be directed to the corresponding authors.
